# A Subtraction-Average-Based Optimizer for Solving Engineering Problems with Applications on TCSC Allocation in Power Systems

**DOI:** 10.3390/biomimetics8040332

**Published:** 2023-07-27

**Authors:** Ghareeb Moustafa, Mohamed A. Tolba, Ali M. El-Rifaie, Ahmed Ginidi, Abdullah M. Shaheen, Slim Abid

**Affiliations:** 1 Electrical Engineerng Department, Jazan University, Jazan 45142, Saudi Arabia; gmoustafa@jazanu.edu.sa (G.M.); smabid@jazanu.edu.sa (S.A.); 2 Electrical Engineerng Department, Suez Canal University, Ismailia 41522, Egypt; 3 Reactors Department, Nuclear Research Center, Egyptian Atomic Energy Authority, Cairo 11787, Egypt; matolba@eaea.org.eg; 4 College of Engineering and Technology, American University of the Middle East, Egaila 54200, Kuwait; 5 Electrical Engineerng Department, Faculty of Engineering, Suez University, Suez 43533, Egypt; ahmed.ginidi@eng.suezuni.edu.eg; 6 Ecole Nationale d’Ingénieurs de Sfax, ENIS Sfax 3038, Tunisia

**Keywords:** benchmark models testing, subtraction-average-based optimizer, cooperative learning technique, allocation problem, power losses minimization

## Abstract

The present study introduces a subtraction-average-based optimization algorithm (SAOA), a unique enhanced evolutionary technique for solving engineering optimization problems. The typical SAOA works by subtracting the average of searcher agents from the position of population members in the search space. To increase searching capabilities, this study proposes an improved SAO (ISAO) that incorporates a cooperative learning technique based on the leader solution. First, after considering testing on different standard mathematical benchmark functions, the proposed ISAOA is assessed in comparison to the standard SAOA. The simulation results declare that the proposed ISAOA establishes great superiority over the standard SAOA. Additionally, the proposed ISAOA is adopted to handle power system applications for Thyristor Controlled Series Capacitor (TCSC) allocation-based losses reduction in electrical power grids. The SAOA and the proposed ISAOA are employed to optimally size the TCSCs and simultaneously select their installed transmission lines. Both are compared to two recent algorithms, the Artificial Ecosystem Optimizer (AEO) and AQuila Algorithm (AQA), and two other effective and well-known algorithms, the Grey Wolf Optimizer (GWO) and Particle Swarm Optimizer (PSO). In three separate case studies, the standard IEEE-30 bus system is used for this purpose while considering varying numbers of TCSC devices that will be deployed. The suggested ISAOA’s simulated implementations claim significant power loss reductions for the three analyzed situations compared to the GWO, AEO, PSO, and AQA.

## 1. Introduction

Optimization is a broad idea used in many sectors of research. An optimization problem constitutes a single issue that possesses more than one viable solution. As a result, the purpose of optimization is to discover the optimal option out of all possible possibilities. The optimization issues are stated mathematically in three parts: objective function, constraints, and decision variables [1]. In the research of optimization, problem solution strategies are classified as deterministic or stochastic [2]. Stochastic techniques solve optimization issues by randomly exploring the searching space and employing arbitrary operators. Such methods build a population of workable solutions to a particular issue before iteratively improving those answers to finally settle on an acceptable solution [3,4,5].

Global optimum is the best response to an optimization issue. Unfortunately, there is no assurance, however, whether the algorithms being used will exactly produce such an optimal solution. As a result, the solution produced by an optimization method for a problem is referred to as a quasi-optimal, which may or may not be equivalent to the global optimum [6]. To organize an effective search in the problem-solving area, metaheuristic techniques must be capable of executing and overseeing queries at the local as well as global levels. The global exploratory investigation leads to an in-depth examination in the space of issue solution and diverts attention from the finest local regions [7]. Local searching, when linked with the concept of exploiting, initiates an exhaustive examination of the most intriguing possibilities for the purpose of converging on possibly better ones. Given the competing goals of discovering and exploiting, establishing an equitable relationship between them throughout the process of searching is critical for the success of metaheuristic techniques [8]. Because of the concept of randomized searching mechanisms, researchers have developed a huge variety of metaheuristic methods. Evolutionary, physics-based, human-based, and swarm-intelligence computational techniques are the four basic types of metaheuristic methods. Evolutionary systems were constructed by representing biological evolutionary qualities such as crossings, mutations, and selections, as detailed in [9,10]. Physical rules drive physics-based techniques, including Archimedes’ optimization algorithm [11], thermal exchange optimization [12], and the equilibrium algorithm (EA) [13,14]. Swarm intelligence computations include the heap-based technique [15], marine predator optimizer [16], grasshopper algorithm [17], jellyfish search optimizer [18], particle swarm optimizer (PSO) [19], artificial bee colony [20], and whale optimization [21]. Several applications are conducted on these optimization frameworks in engineering fields, especially regarding renewable energy. In [22], the ant colony optimizer (ACO) has been intended for training a multilayer feed-forward neural network control system to derive the maximum power point tracking (MPPT) from photovoltaic (PV) arrays supplying an arc welding machine (AWM). In [23], a PSO has been enhanced with a Gauss mapping chaotic component and combined with MPPT for a wind conversion system based on a permanent magnet (PM) synchronous generator to run its tip-to-speed ratio at the optimal level. In [24,25] the ACO algorithm has been adopted to optimally find the tuning parameters of the fractional order proportional integral derivative (FOPID) controller of a PMDC motor that drive a wire-feeder system (WFS) regarding AWM. In [26], a PSO algorithm has been integrated with an MPPT controller depending on the adaptive neuro fuzzy inference system to track the maximum available power from a PV system that utilized for supplying WFS of AWM with varying weather conditions. For dealing with diverse engineering design challenges, an improved artificial ecosystem optimizer has been conducted [27]. The amalgamated fitness-distance architecture facilitates in identifying people who effectively improve the solution quality which was designed to handle engineering design tasks such as hydrostatic thrust bearing, planetary gear train, speed reducer, pressure vessel, and rolling element bearing.

Recently, a technique named SAOA [1] has been presented where its fundamental premise is to update population members’ locations in the search space by deducting the average of searcher agents. This technique is beneficial since it can be easily applied to engineering applications and has minimal parameters that need to be changed. The results of the SAOA were contrasted with more modern approaches and other existing techniques considering several benchmark models [1]. In this paper, an ISAOA is presented to enhance the searching capability. The proposed ISAOA augments the standard SAOA, including a cooperative learning strategy depending on the leader solution. Additionally, the proposed ISAOA is adopted to handle power system applications for Thyristor Controlled Series Capacitor (TCSC) allocation-based loss reductions in electrical power grids.

In order to meet load demand through power export-import agreements, utilities heavily rely on present generation capacity because the installed generating units are often situated distant from load centers. As a result, actual power systems include several connections. The minimization of power system losses is a pivotal technical objective function to enhance the voltage quality for all common coupling system points. It can be optimized using the optimal power flow issue (OPFI) [28], reactive power management [29], ancillary services [30], and flexible alternating current transmission systems (FACTS) devices incorporation [31]. For such supporting strategies, maintaining load flow balance and keeping operating variables within the associated constraints are necessary, including transmission network limitations, voltage restrictions, generator output limitations, and valve restrictions [32]. Researchers have created a number of traditional and metaheuristic optimization methods recently in an effort to solve the OPFI [33]. The traditional approaches include the sequential unconstrained minimization technique [34], interior point approach [35], linear and nonlinear programming, gradient methods, interior-point methods, Newton method, Newton-based method [36], and fuzzy linear frameworks. However, it has been noted that these methods cannot be used for large power systems and do not produce global ideal solutions. Therefore, researchers have attempted to develop metaheuristic methods to sidestep the issues that traditional methods possess. There are diverse population-based heuristics that are used to solve the OPFI, such as the electromagnetism-like mechanism [37], simulated annealing optimization [38], Particle Swarm Optimization (PSO) [39], Gradient-Based Optimization Algorithm (GBOA) [40], and Quantum computing with Moth Flame Technique (QMFT) [41]. In addition, in [42], the TLBO technique has been developed and adopted for solving the allocation optimization problem in power systems of capacitors for the sake of power factor correction. To suitably increase the incorporation of dispersed sources of energy in low-inertia electrical networks, an updated priority-list approach with a Boolean inference coding/decoding method and a feed-forward neural network for determining the subsequent function assessment was hybridized [43]. Priority-based dynamic computing was used in this study to solve the unit commitment issue by simulating various scenarios with growing renewable energy. In [44], a priority list approach based on a genetic algorithm has been presented for investigating the expansion problem of intermittent renewable sources and measuring their effects on the total cost of production, involving renewable generation curtailment and load shedding avoidance while taking into account various types of electricity storage. In [45], Bayesian optimization was adopted with Gaussian process regression for finding the best unit commitment scheduling for coping with the variable and fluctuating behavior of energy from renewable sources. In [46], the Gorilla Troops Technique (GTT) has been employed on the OPFI with IEEE 30 bus system. The GTT includes five strategies for the group behaviors of gorillas which are visiting other gorillas, migrating to a new area, migrating in a certain direction, vying for adult females, and following the silverback. In [47], GTT has been employed on the OPFI with the inclusion of TCSC devices in the system. However, the size and allocation of the TCSC have not been taken into consideration. An Emended Crow Search Algorithm (ECSA) has been employed on the OPFI as depicted in [48] with adjustments to aggregated novel bat algorithm. For the purpose of reducing the costs of energy losses with/without the inclusion of voltage-source-converter stations, a manta-ray foraging optimizer has been designed for electrical grids in Ref. [49]. 

The significant contributions mentioned in this study are listed below.

- A novel ISAOA is presented.
- The proposed ISAOA establishes great superiority over the standard SAOA after considering testing on different standard mathematical benchmark functions.
- Compared to existing studies, the placement and sizing of TCSC devices are handled to minimize power losses.
- In this context, considering the standard IEEE 30 bus power system, the proposed ISAOA outperforms various SAOA and other recent approaches of GWO, AEO, PSO, and AQA.
- Considering different numbers of TCSC devices, the suggested ISAOA’s precision and quality of solution are demonstrated compared to the others.

## 2. Novel ISAOA Version: Mathematical Model 

This section explains the theory behind the ISAOA technique that has been suggested and a presentation of its mathematical modeling for use in optimization problems.

### 2.1. Standard SAOA Version

The basic idea behind the standard SAOA is to update the location of population members in the search space by subtracting the average of searcher agents [1]. The search space represents the name given to the set of solutions to each optimization issue. The dimension length of the search space is the number of control variables in the problem being studied. Their numerical values are determined by the positions of the algorithm’s searching individuals consisting of the population size. As a result, every seeking solution or individual is computationally represented employing a vector and comprises data pertaining to the control variables. Random initialization determines the search agents’ initial principal locations in the search space as follows [1]:

(1) $$Sa_{i}=LL+Range\times rand(1,Dim)\;i=1:Ns$$

where *Sa_i_* indicates a solution vector agent in the SAOA population, which has a size of solution (*Ns*). *Dim* symbolizes the dimensional length regarding the number of control variables. *LL* indicates the lower limit of the control variables. *Range* denotes an acceptable interval of the dimensions, which can be expressed as follows [1]:

(2) $$Range_{i}=UL_{i}-LL_{i}\;i=1:Dim$$

where *UL* indicates the higher limit of the control variables. 

Each individual investigation seems to be a potential solution to the considered optimization aspect. Therefore, based on each search individual, the problem’s objective function can be assessed. *Fit_i_* can be used to represent the evaluated values for the problem’s objective function for each solution vector agent (*i*). According to the assessed values for the objective function, the optimal solution is determined using the best value generated for the goal function. In addition, the worst solution corresponds to the worst value determined for the goal function. 

Mathematical considerations, including mean values, variations in search representative placements, and the sign of the variation between two objective values, served as the foundation for the SAOA’s conception. Because it relies on a special functioning “v” known as the “v-subtraction operator,” the SAOA method for computing the arithmetic mean is wholly original. Therefore, each solution vector agent in the SAOA population is updated related to the following equation [1]:

(3) $$Sa_{i,new}=Sa_{i}+\vec{z_{i}}\times \frac{1}{Ns}\left(\sum_{k=1}^{Ns}\left(Sa_{i}{-}_{\upsilon }Sa_{k}\right)\right)\;i=1:Ns$$

where *Sa_i,new_* indicates the new upgraded solution vector agent in the SAOA population. $\vec{z_{i}}$ denotes a vector of size *Dim*, containing numbers inside the range [0, 1] and a normal distribution for each of its elements. $Sa_{i}{-}_{\upsilon }Sa_{k}$ indicates the subtraction operation of the two searching solutions (*Sa_i_* and *Sa_k_*) from the SAOA population, which can be mathematically represented as follows [1]:

(4) $$Sa_{i}{-}_{\upsilon }Sa_{k}=sign\left(Fit_{i}(Sa_{i})-Fit_{k}(Sa_{k})\right)\left(Sa_{i}-\vec{\upsilon }⊙Sa_{k}\right)$$

where ⊙ denotes the Hadamard product symbol. $\vec{\upsilon }$ is a randomized vector of size *Dim* containing numbers from the range [1, 2]. *Fit_i_(Sa_i_)* and *Fit_k_(Sa_k_)* are the evaluated objective values for solutions (*i*) and (*k*), respectively.

After updating each solution vector, the objective value is estimated and assessed. Then, in accordance with (5), this newly created solution replaces the old one if the new solution provides a better objective score as follows [1]:

(5) $$Sa_{i}=\{\begin{array}{c} Sa_{i,new}\\ Sa_{i} \end{array}\begin{array}{c} if\;Fit_{i,new}(Sa_{i,new})\leq Fit_{i}(Sa_{i})\\ Else \end{array}$$

### 2.2. Novel ISAOA Version Incorporating a Cooperative Learning Strategy

In an effort to enhance the searching capability, an ISAOA is proposed in this study by incorporating a cooperative learning strategy depending on the leader solution. In the standard SAOA version, as described in the updating mechanism of Equation (3), the change in position of every searching solution (*Sa_i_*) within the search space is determined by the arithmetic mean of the v-subtraction operator of all the other solution vectors in the population, from it. Using this framework, the exploration characteristics are significant and powerful. On the other side, the exploitation searching characteristics require further enhancement by supporting the local searching mechanism around the best promising area. In order to accomplish that, a cooperative learning strategy is merged in the ISAOA version to provide learning information from the best solution vector as follows:

(6) $$Sa_{i,new}=Sa_{BEST}+\vec{w_{i}}\times \left(Sa_{R1}-Sa_{R2}\right)$$

where $Sa_{i,new}$ indicates the new upgraded solution vector agent in the SAOA population. $Sa_{BEST}$ refers to the best solution in the current iteration; $\vec{w_{i}}$ denotes a vector of size *Dim*, containing numbers inside range [0, 1] and a normal distribution for each of its elements. $Sa_{R1}$ and $Sa_{R2}$ represent two random unequal picked from the SAOA population.

**Figure 1:**
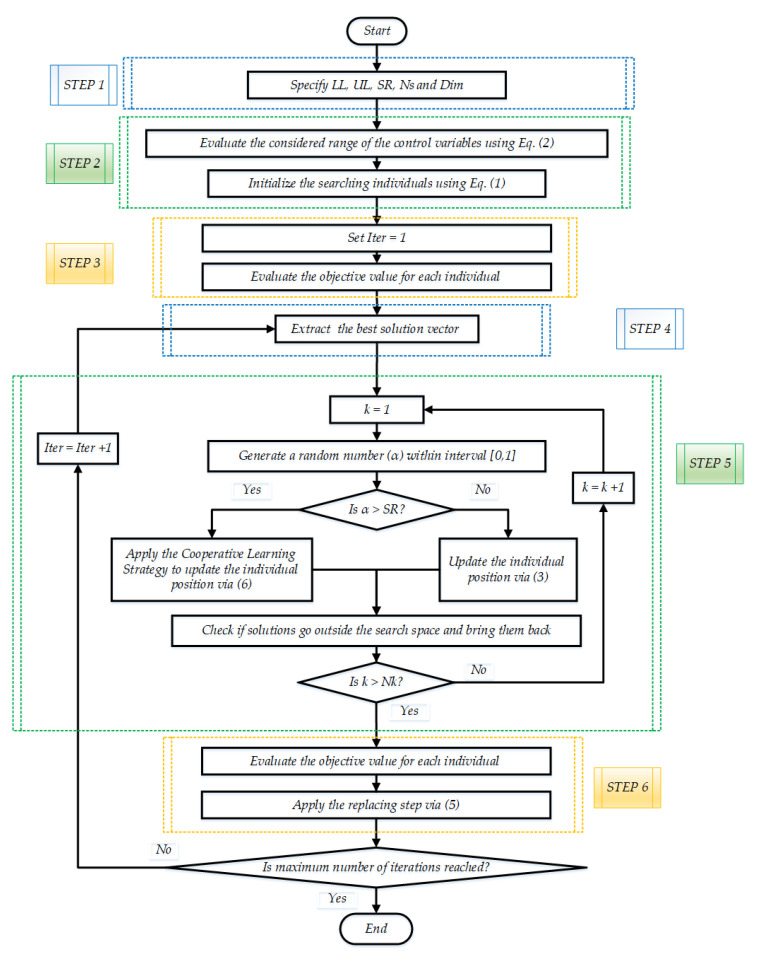
Key phases of the suggested ISAOA.

In order to provide a balance between the exploration characteristics described in Equation (3) and the augmented exploitation characteristics described in Equation (6), a selection probability (SR) is preserved, which is set to 50%. The abovementioned key phases of the suggested ISAOA are shown in Figure 1.

## 3. Experimental Validation of Standard Benchmarking Functions

In this section, an evaluation of the formed ISAOA and the standard SAOA is made in comparison to ten popular, well-known mathematical benchmarks that are listed in Table 1 [50]. While the second function (F2) is multimodal, the first function (F1) represents a unimodal function. Mixed functions are represented by functions (F3–F6), while composite functions are represented by functions (F7–F10). According to the no free lunch theorem, no optimization algorithm is the best for all optimization problems. Therefore, the comparisons involve several problems with distinct features. These simulations were carried out using MATLAB 2017b software. 

**Figure 2:**
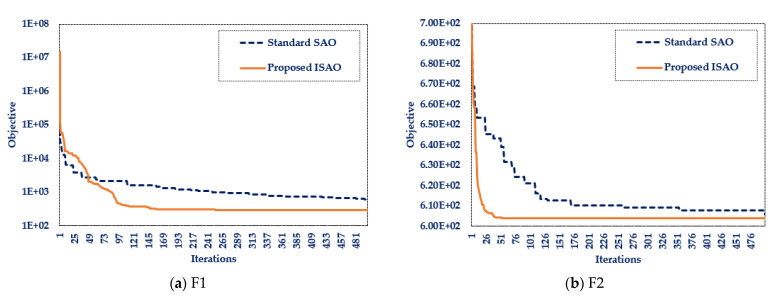

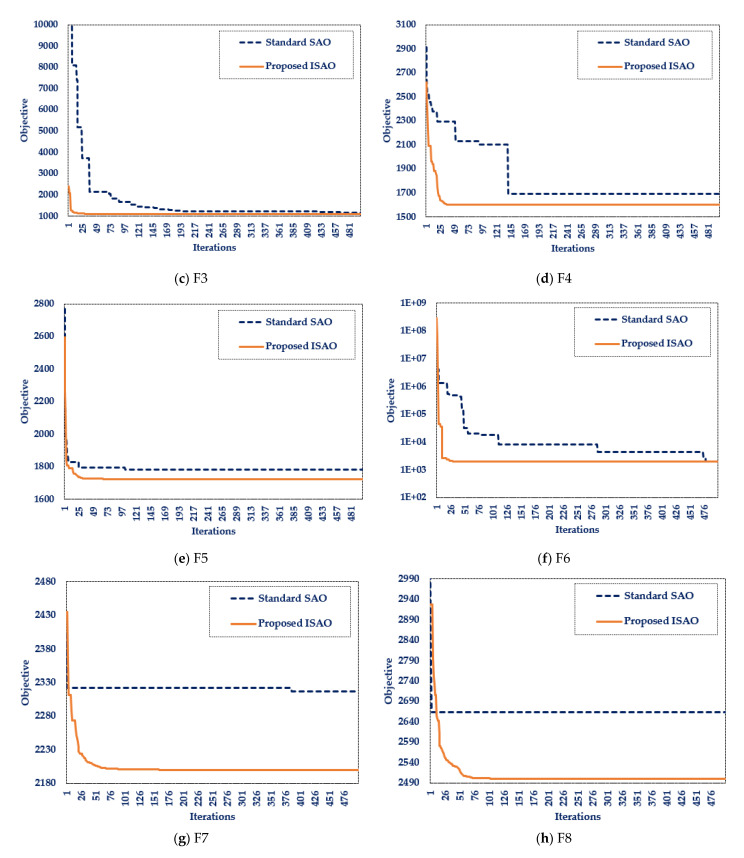

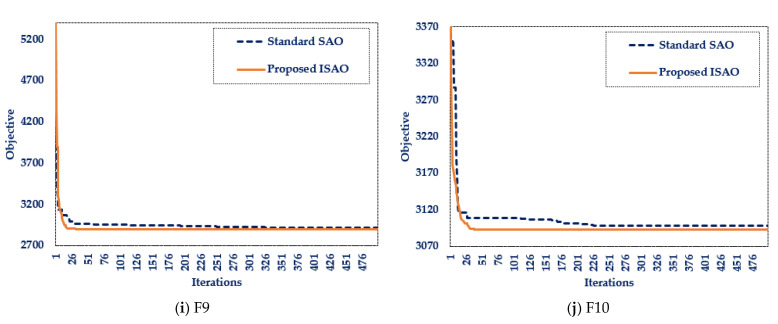
Benchmarks’ best convergence curves for the designed ISAOA and standard SAOA.

The performance analysis of the designed ISAOA and standard SAOA for ten widely used mathematical problems is shown in Table 2. Additionally, Figure 2 presents the most desirable convergent motion characteristics. This table demonstrates the resilience of the produced ISAOA in determining the optimal solution to the majority of the investigated mathematical problems by explicitly showing that the designed ISAOA runs and works better than the standard SAOA in the tested mathematical functions. According to the best-obtained objective, the suggested ISAOA outperforms the standard SAOA for all investigated benchmarks except F6, with a success rate of 90%. In addition, the suggested ISAOA surpasses the regular SAOA for all examined benchmarks, with the exception of F6 and F10, according to the mean achieved objective, with a success rate of 80%. Moreover, the adopted ISAOA surpasses the regular SAOA for all benchmarks examined, with the exception of F6, F8, and F10, with a success rate of 70%, according to the worst attained objective. According to this table, the proposed ISAOA beats the conventional SAOA for the best, mean, worst, and standard deviations in 70% of the benchmark functions’ statistical indices regarding the benchmarks investigated. The success rates attested demonstrate the considerable efficacy of the proposed ISAOA combining the advised cooperative learning technique.

## 4. TCSC Allocation-Based Loss Minimization in Electrical Power Grids

**Figure 3:**
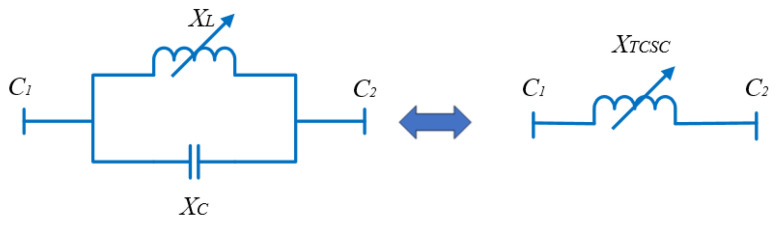
TCSC modeling.

One of the most well-known FACTS devices in the series is the TCSC, which has a number of benefits, including great performance, rapid response, and low cost. Inductive and capacitive capability compensations are the two reactive operational modes available for TCSC devices. The reactance of the corresponding transmission line can be, consequently, increased or decreased in both modes. TCSC modeling in electrical systems connected in series with a line is shown in Figure 3. It is composed of a capacitance (C) coupled in parallel with an inductance (L), which is modulated by a valve located in anti-parallel conventional thyristors [51,52,53,54].

In order to technically enhance the power grid and the voltage quality in all buses, the primary objective is to minimize the whole grid losses, which can be mathematically represented as illustrated [55]:

(7) $$\mathrm{OJV}=\sum_{p=1}^{\mathrm{Npq}}\sum_{p\neq q}^{\mathrm{Npq}}G_{pq}{(V}_{p}{}^{2}{+V}_{q}{}^{2}{-2(V}_{p}V_{q}{\cos\;(\theta }_{p}{-\theta }_{q}))$$

where *N_bq_* manifests the number of buses; *G_pq_* points out the transfer conductance among buses *p* and *q*; *θ* signifies the phase angle; *V* represents the voltage. 

To address the TCSC allocation problem, several equality and inequality limitations must be maintained, which are related to control and dependent variables. First, regarding the control variables, the TCSC locations and the regarding reactance compensation have to be satisfied as illustrated in Equations (8) and (9), respectively.

(8) $$1\leq Line_{TCSC,p}\leq N_{lines},\;\forall \;p\in \left[1,N_{TCSC}\right]$$

(9) $$0.5\times X_{Line_{TCSC,p}}\leq X_{TCSC}{\left(\alpha \right)}_{p}\leq -0.5\times X_{Line_{TCSC,p}},\;\forall \;p\in \left[1,N_{TCSC}\right]$$

where *Line_TCSC,p_* manifests the candidate lines to install TCSC devices; *N_lines_* points out the whole number of lines; *N_TCSC_* signifies the number of TCSC devices to be installed; $X_{Line_{TCSC,p}}$ represents the reactance of the regarding lines selected to install TCSC devices. 

The TCSC devices are sized to ensure the capacitor bank compensates for 50–70% of the transmission line. Therefore, the maximum limit of capacitor bank compensations is considered 50%, as displayed in Equation (9) which is based on several other previously published articles [46,47,51,52,53,54].

In addition, regarding the control variables, the limitations for the output powers from generators, generators voltage, reactive powers injection from Var sources, and tap settings are handled using Equations (10)–(13), respectively [56,57].

(10) $${\mathrm{Pgen}}_{p}^{\min}\leq {\mathrm{Pgen}}_{p}\leq {\mathrm{Pgen}}_{p}^{\max},\;\forall \;p\in \left[1,Ngen\right]$$

(11) $${\mathrm{Vgen}}_{{}_{p}}^{\min}\leq {\mathrm{Vgen}}_{{}_{p}}\leq {\mathrm{Vgen}}_{p}^{\max},\;\forall \;p\in \left[1,Ngen\right]$$

(12) $${\mathrm{Qc}}_{\mathrm{VAR}}^{\min}\leq {\mathrm{Qc}}_{\mathrm{VAR}}\leq {\mathrm{Qc}}_{\mathrm{VAR}}^{\max},\;\forall \;\mathrm{VAR}\in \left[1,Nq\right]$$

(13) $${\mathrm{Tp}}_{\mathrm{Tr}}^{\min}\leq {\mathrm{Tp}}_{\mathrm{Tr}}\leq {\mathrm{Tp}}_{\mathrm{Tr}}^{\max},\;\forall \;\mathrm{Tr}\in \left[1,Nt\right]$$

where *Ngen* represents the total number of generation units; *Nt* illustrates the total number of transformer units; *Nq* characterizes the total number of compensating/capacitors units; *Pgen* illustrates generators’ real power output; *Tp* denotes the tap changer settings; *Vgen* gives voltages of the generators; *Qc* signifies the reactive power injections of switching reactors/capacitors.

Second, regarding the dependent variables, the limitations for the output reactive powers from generators, power flow through the lines, and load bus voltages are handled using Equations (14)–(16), respectively [55].

(14) $${\mathrm{Qgen}}_{{}_{p}}^{\min}\leq {\mathrm{Qgen}}_{{}_{p}}\leq {\mathrm{Qgen}}_{{}_{p}}^{\max},\;\forall \;p\in \left[1,Ngen\right]$$

(15) $$\left|{\mathrm{Sfl}}_{\mathrm{Line}}\right|\leq {\mathrm{Sfl}}_{\mathrm{Line}}^{\max},\;\forall \;\mathrm{Line}\in \left[1,N_{lines}\right]$$

(16) $${\mathrm{VL}}_{j}^{\min}\leq {\mathrm{VL}}_{j}\leq {\mathrm{VL}}_{j}^{\max},\;\forall \;j\in \left[1,NPQ\right]$$

where *Qgen* characterizes generator reactive power outputs; *Sfl* elaborates transmission flow limits; *VL* shows load bus voltage magnitudes at bus *j*; *NPQ* signifies the entire number of load buses.

On the other side, the active and reactive power load balancing equations at each bus must be maintained as equality constraints. These constraints are fully achieved with the convergence of the load flow routine.

## 5. Simulation Results for Optimal TCSC Allocations in Power Systems

**Figure 4:**
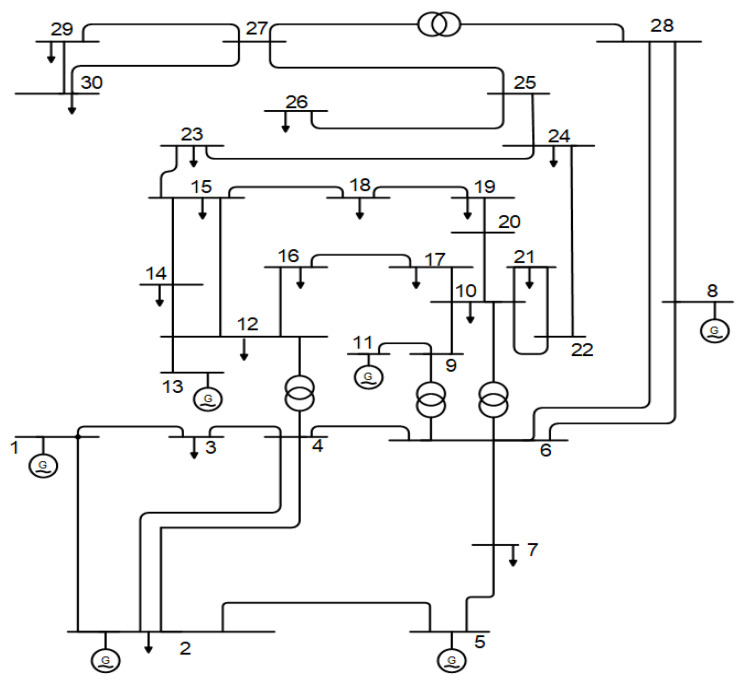
Schematic diagram of IEEE 30 bus grid [65].

The proposed ISAOA is applied in this section to solve the considered problem of optimal TCSC allocations in power systems to minimize power losses. In this regard, the IEEE standard 30 bus system is considered. The IEEE 30 bus grid is displayed in Figure 4 [58], which consists of 41 branches, 30 buses, 4 tap transformers, and 9 VAr compensators. The whole data set for the generation limitations, lines, and buses are derived from [59]. The maximum generator voltages and tap positions correspond to 1.1 and 0.9 p.u. For load buses, the maximum voltage remains 1.05 and 0.95 p.u., correspondingly. The limits for the generator voltage and tap settings are 1.1000 and 0.9000 p.u., respectively. The voltage limits of load buses are considered to be 1.0500 and 0.9500 p.u., respectively. The proposed ISAOA is contrasted with the standard SAOA and other recent algorithms, which demonstrated previous effective applications such as AEO [60], AQA [61,62], PSO, and GWO [63,64]. The five compared algorithms are applied with the same 50 searching individuals and 300 iterations. They are performed 20 separate times considering three different case studies as follows:

- Case 1: One TCSC to be allocated.
- Case 2: Two TCSCs to be allocated.
- Case 3: Three TCSCs to be allocated.

### 5.1. Application for Case 1

In this case, one TCSC device must be assigned to the power grid by looking for the best location and size. To reduce power losses, the suggested ISAOA is used in place of the standard SAOA, AEO, AQA, PSO, and GWO. 

Table 3 tabulates the optimal control variables related to the compared results in terms of the generator’s voltage and output power, the Var sources injection power, and the tap value, in addition to the placement and sizing of the TCSC device. As illustrated, the suggested ISAOA yields the lowest power losses of 2.8217 MW with the best performance. The proposed ISAOA selects the transmission lines (28-27) with a compensation level of approximately 50% subtraction from the installed line reactance. 

According to the proposed ISAOA, the achieved power losses represent a significant reduction percentage of 51.62% compared to the initial case. On the other side, the standard SAOA obtains power losses of 3.061 MW upon installing a TCSC device in series with lines (23-24) with a compensation level of approximately 4.66% addition. The proposed ISAOA derives a significant reduction percentage of 7.81% compared to the SAOA. Additionally, the AEO acquires power losses of 2.884 MW upon connecting a TCSC device in series with lines (28-27) with a compensation level of roughly 49.49% subtraction. Upon adding a TCSC device in series with lines (4-6) with a compensation level of around 35.03% subtraction, the GWO also achieves power losses of 3.035 MW. Additionally, upon connecting a TCSC device in series with lines (6-28) and subtracting a compensation level of roughly 42.017%, the AQA achieves power losses of 2.99 MW. As shown in Table 3, the proposed ISAOA derives the best performance upon acquiring the smallest measurement of the best power losses of 2.8217 MW, respectively. On the other side, the SAOA, AEO, GWO, PSO, and AQA achieve losses of 3.061, 2.844, 3.035, 3.37, and 2.990 MW, respectively.

**Figure 5:**
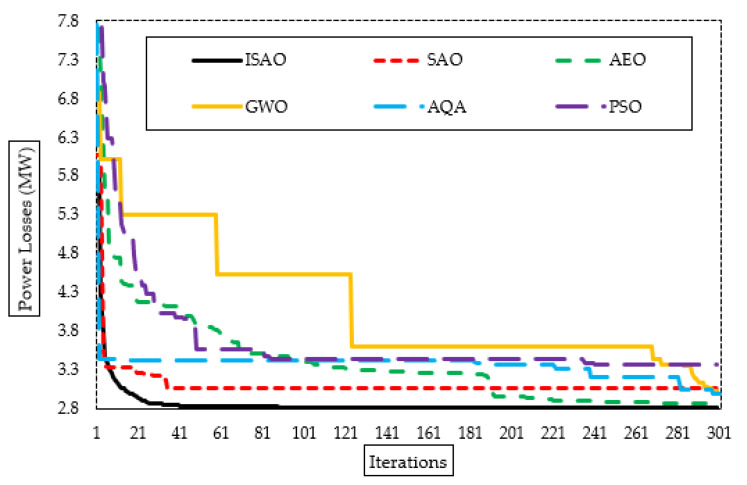
Convergence characteristics of suggested ISAOA, standard SAOA, AEO, AQA, PSO, and GWO for Case 1.

The suggested ISAOA, standard SAOA, AEO, AQA, PSO, and GWO converging properties are also shown in Figure 5. As shown, the proposed ISAOA has a high and quick capability to find promising areas. After only fifty iterations, it starts, with precedence compared to the others, searching to minimize the losses of less than 2.83 MW. Despite the AQA converging at an earlier iteration, it becomes stuck in a local optimizing area for more than 150 iterations.

**Figure 6:**
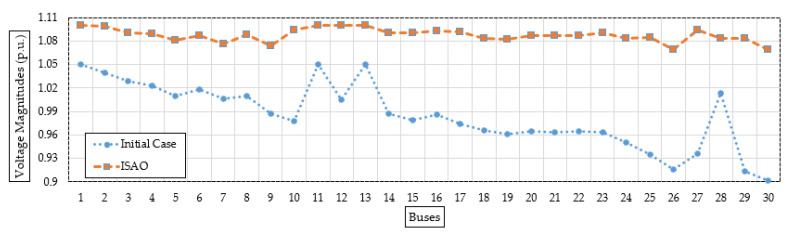
Bus voltages after installing the candidate TCSC device (Case 1) based on the proposed ISAOA versus the initial case.

Figure 6 shows the bus voltage after installing the candidate TCSC device based on the suggested ISAOA vs. the initial instance to demonstrate a range of voltage enhancements. As can be seen, grid buses have made significant advancements. The last grid bus (No. 30) has the biggest voltage profile rise, increasing from 0.9012 per unit (p.u.) to 1.0696 p.u. with an improvement percentage of 15.74%.

**Figure 7:**
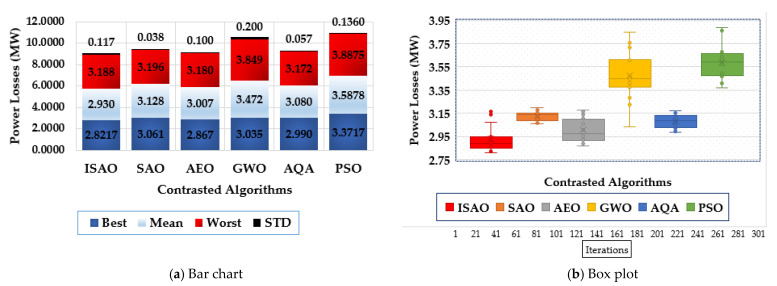
Statistical characteristics of suggested ISAOA, standard SAOA, AEO, AQA, PSO, and GWO for Case 1.

To assess the statistical evaluation of the compared techniques, Figure 7 displays the characteristics of the suggested ISAOA, standard SAOA, AEO, AQA, PSO, and GWO in terms of the best, mean, worst and standard deviation (STD) over the separate runs. As shown, the proposed ISAOA derives the best performance by acquiring the smallest measurements of the best and mean power losses of 2.8217 and 2.93 MW, respectively.

### 5.2. Application for Case 2

Upon choosing the appropriate position and size, two TCSC devices may be connected to the power grid. The proposed ISAOA is employed in place of the conventional SAOA, AEO, AQA, PSO, and GWO in order to minimize power losses. The associated appropriate control variables are shown in Table 4. As shown, the recommended ISAOA produces the highest performance and the lowest power losses of 2.82 MW. It chooses the transmission lines (28-27) and (6-28) with 50% subtraction compensation levels. The conventional SAOA, AEO, GWO, PSO, and AQA, on the other hand, experience power losses of 3.102, 2.867, 3.227, 3.36, and 2.995 MW, respectively. 

**Figure 8:**
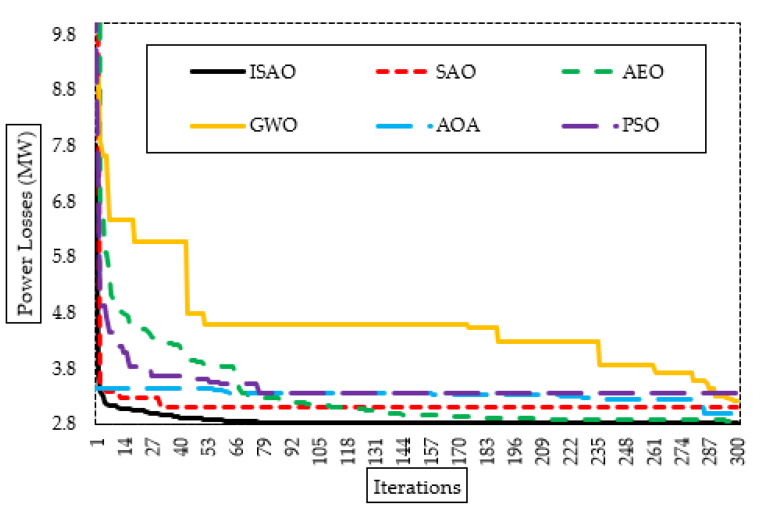
Convergence characteristics of suggested ISAOA, standard SAOA, AEO, AQA, PSO, and GWO for Case 2.

**Figure 9:**
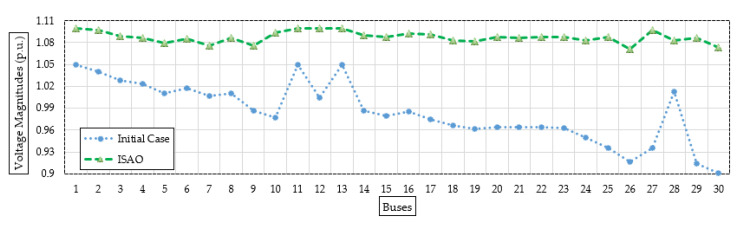
Bus voltages after installing the candidate TCSC device (Case 2) based on the proposed ISAOA versus the initial case.

Figure 8 also displays the proposed ISAOA, standard SAOA, AEO, AQA, PSO, and GWO convergence features. As demonstrated, the suggested ISAOA is highly and quickly capable of identifying interesting locations. After just 75 iterations, it searches for a reduction in power loss below 2.83 MW with primacy over the others. In addition, Figure 9 shows the bus voltage after installing the candidate TCSC devices based on the suggested ISAOA vs. the initial instance to demonstrate the range of voltage enhancements. As can be seen, grid buses have made significant advancements.

**Figure 10:**
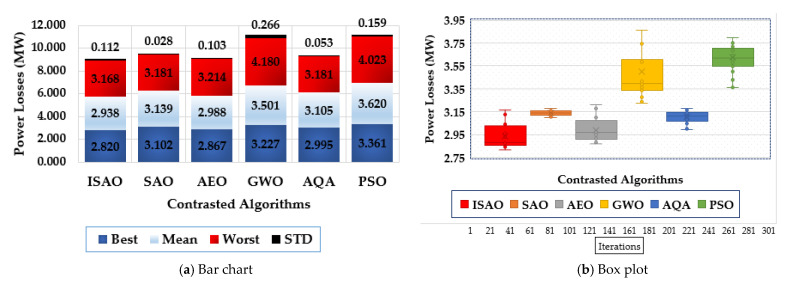
Statistical characteristics of suggested ISAOA, standard SAOA, AEO, AQA, PSO, and GWO for Case 2.

Figure 10 shows the features of the proposed ISAOA, conventional SAOA, AEO, AQA, PSO, and GWO across the individual runs to evaluate their statistical assessment. It has been demonstrated that the suggested ISAOA outperforms the competition. According to the best losses, the proposed ISAOA acquires 2.82 MW losses with an improvement of 9.08%, 1.62%, 12.60%, 16.1%, and 5.85%, respectively compared to the standards SAOA, AEO, AQA, PSO, and GWO. In addition, the suggested ISAOA, when compared to the normal SAOA, AEO, AQA, PSO, and GWO, acquires 2.938 MW losses with improvements of 6.39%, 1.66%, 16.08%, 18.8%, and 5.37%, respectively, in line with the mean losses. The designed ISAOA acquires 3.168 MW losses in accordance with the worst losses, with improvements of 0.41%, 1.43%, 24.21%, 21.3%, and 0.40%, respectively, compared to the standard SAOA, AEO, AQA, PSO, and GWO.

### 5.3. Application for Case 3

In this case, three TCSC devices may be connected to the electrical grid upon selecting the right position and size. In order to reduce power losses, the suggested ISAOA is used in place of the traditional SAOA, AEO, AQA, PSO, and GWO. Table 5 displays the associated suitable control variables. As can be seen, the suggested ISAOA yields the best results and the lowest power loss of 2.821 MW. It selects the transmission lines (6-28), (10-20), and (28-27) with subtraction compensation values of 36.96%, 50%, and 50%, respectively. On the other hand, the standard SAOA, AEO, GWO, PSO, and AQA suffer power losses of 3.036, 2.880, 3.187, 3.328, and 2.969 MW, respectively.

**Figure 11:**
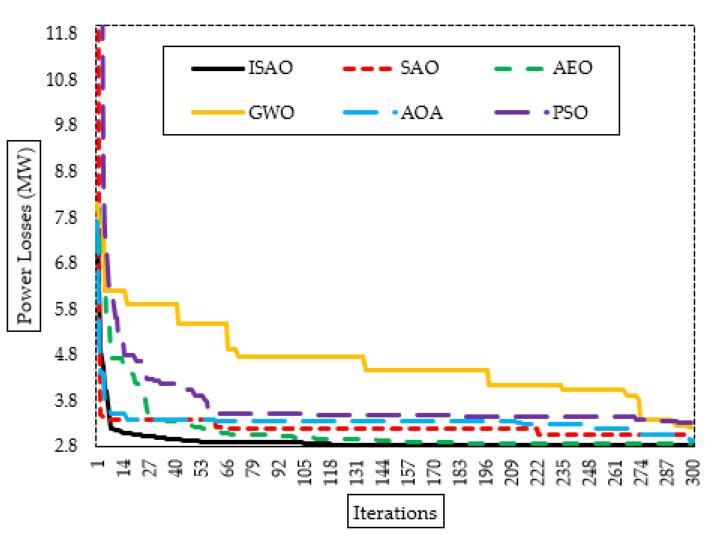
Convergence characteristics of suggested ISAOA, standard SAOA, AEO, AQA, PSO, and GWO for Case 3.

Figure 11 additionally shows the proposed ISAOA, standard SAOA, AEO, AQA, PSO, and GWO convergence features. As demonstrated, the suggested ISAOA is highly and quickly capable of identifying interesting locations. After just 79 iterations, it begins to hunt to decrease the losses below 2.83 MW with precedence over the others.

**Figure 12:**
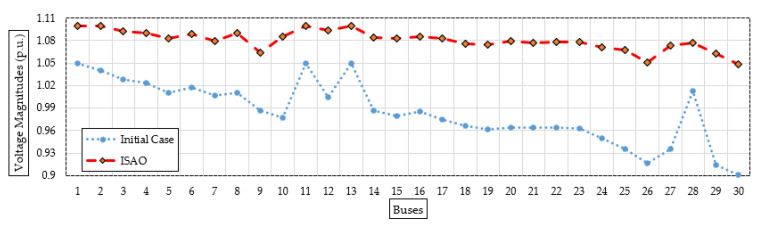
Bus voltages after installing the candidate TCSC device (Case 3) based on the proposed ISAOA versus the initial case.

**Figure 13:**
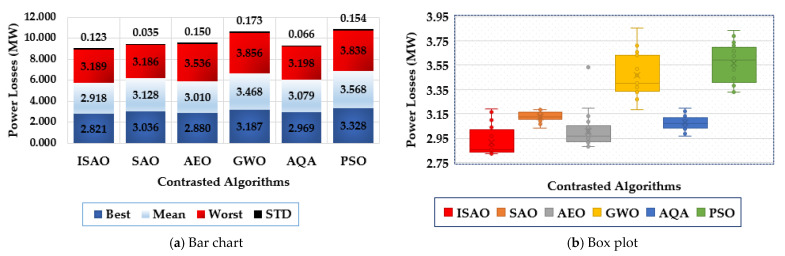
Statistical characteristics of suggested ISAOA, standard SAOA, AEO, AQA, PSO, and GWO for Case 3.

To illustrate the range of voltage enhancement, Figure 12 compares the bus voltages before and after installing the proposed TCSC devices based on the indicated ISAOA. Grid buses have progressed significantly, as is evident. The statistical evaluation of the proposed ISAOA, standard SAOA, AEO, AQA, PSO, and GWO is also shown in Figure 13 in terms of the best, mean, worst, and standard deviation (STD) over the individual runs. As it has been demonstrated, the suggested ISAOA achieves the greatest performance by gaining the smallest measurements for the best and mean worst losses, which are, respectively, 2.821 and 2.918. 

### 5.4. Analysis of Increasing the Maximum Compensation Level to 70%

As previously formulated in Equation (9), the TCSC reactance compensation has to be satisfied and limited to 50%. In this section, an analysis of increasing the maximum compensation level to 70% is investigated instead of Equation (9) considering the following constraint:

(17) $$0.7\times X_{Line_{TCSC,p}}\leq X_{TCSC}{\left(\alpha \right)}_{p}\leq -0.7\times X_{Line_{TCSC,p}},\;\forall \;p\in \left[1,N_{TCSC}\right]$$

For this analysis, to reduce power losses, the suggested ISAOA is used, considering maximum compensation levels of 50% and 70%, respectively. The three cases are investigated based on the number of TCSC devices to be allocated, and the related results are stated in Table 6. 

As shown, considering the 70% compensation limit, the proposed ISAOA achieves power losses of 2.80645 and 2.775199 for Cases 2 and 3, respectively. On the other side, considering the 50% compensation limit, the proposed ISAOA achieves power losses of 2.820 and 2.821 for Cases 2 and 3, respectively. This output assessment shows more compensation derives more reduction in power losses. The compensation of 70% derives a reduction in power losses of 0.5% and 1.62% for Cases 2 and 3, respectively.

## 6. Conclusions

This study proposes a novel, improved evolutionary method dubbed the Subtraction-Average-based optimization algorithm. The proposed ISAOA incorporates a cooperative learning strategy depending on the leader solution to enhance the searching capability. The proposed ISAOA shows great superiority in enhancement compared with the standard SAOA with experimental validation of 10 standard benchmarking functions. It beats the conventional SAOA for the best, mean, worst, and standard deviation measurements in 70% of the benchmark functions’ statistical indices regarding the benchmarks investigated. The success rates attested demonstrate the considerable efficacy of the proposed ISAOA combining the advised cooperative learning technique. Further, the TCSC allocation-based loss reduction in electrical power grids is achieved through the development of the ISAOA algorithm. In comparison to the conventional SOA, GWO, AEO, PSO, and AQA, the suggested ISAOA is successfully implemented for the IEEE-30 bus power grid standard. The suggested ISAOA’s simulated implementations assert significant power loss reductions for the three examined cases studied compared to the others. On the basis of the suggested ISAOA for all grid buses, significant improvement is also gained.

The proposed ISAOA derives the best performance by acquiring the smallest measurements of the best power losses of 2.8217, 2.82, and 2.821 MW, respectively, for the three cases studied. In addition, the voltage profiles of all buses are enhanced with an improvement percentage of 15.74%. As a future area of study, the application of more advanced algorithms can be developed and applied with statistical comparisons in order to find highly robust and superior solution quality.

## Figures and Tables

**Table 1:** Definitions in detail of the 10 prevalent benchmarks being considered [50].

Function No.	Function	Dim	Max.	Min.	Optimal
F1	Shifted and Rotated Zakharov Function	30	100	−100	300
F2	Shifted and Rotated Expanded Scaffer’s F6 Function	30	100	−100	600
F3	Hybrid Function 1 (N = 3)	30	100	−100	1100
F4	Hybrid Function 6 (N = 4)	30	100	−100	1600
F5	Hybrid Function 6 (N = 5)	30	100	−100	1700
F6	Hybrid Function 6 (N = 5)	30	100	−100	1900
F7	Composition Function 1 (N = 3)	30	100	−100	2100
F8	Composition Function 4 (N = 4)	30	100	−100	2400
F9	Composition Function 5 (N = 5)	30	100	−100	2500
F10	Composition Function 7 (N = 6)	30	100	−100	2700

**Table 2:** Performance study for the designed ISAOA and standard SAOA on different benchmarks.

Function	Index	Algorithms		Proposed ISAOA vs. Standard SAOA	Improvement Percentage (%)	Decline Percentage (%)
		Standard SAOA	Proposed ISAOA			
F1	Best	623.6	300.0	√	51.9%	-
	Mean	2872.2	300.0	√	89.6%	-
	Worst	8350.7	300.3	√	96.4%	-
	STD	1682.4	0.0	√	100.0%	-
F2	Best	606.0	603.8	√	0.4%	-
	Mean	620.6	617.5	√	0.5%	-
	Worst	649.2	647.2	√	0.3%	-
	STD	10.3	9.4	√	8.4%	-
F3	Best	1166.7	1103.3	√	5.4%	-
	Mean	1493.4	1158.7	√	22.4%	-
	Worst	2561.0	1273.3	√	50.3%	-
	STD	372.1	43.4	√	88.3%	-
F4	Best	1694.8	1601.7	√	5.5%	-
	Mean	2077.5	1817.6	√	12.5%	-
	Worst	2315.7	2135.6	√	7.8%	-
	STD	119.4	142.0	x	-	15.9%
F5	Best	1783.3	1724.0	√	3.3%	-
	Mean	1888.6	1784.0	√	5.5%	-
	Worst	2094.9	1925.6	√	8.1%	-
	STD	80.5	53.5	√	33.5%	-
F6	Best	2022.8	2025.1	x	-	0.1%
	Mean	9198.0	11852.1	x	-	22.4%
	Worst	32007.5	208941.3	x	-	84.7%
	STD	6583.5	28897.4	x	-	77.2%
F7	Best	2316.5	2200.0	√	5.0%	-
	Mean	2347.7	2311.1	√	1.6%	-
	Worst	2385.1	2379.0	√	0.3%	-
	STD	13.0	53.6	x	-	75.8%
F8	Best	2663.2	2500.0	√	6.1%	-
	Mean	2777.7	2776.8	√	0.0%	-
	Worst	2859.1	2881.8	x	-	0.8%
	STD	28.1	48.7	x	-	42.1%
F9	Best	2918.9	2897.9	√	0.7%	-
	Mean	2959.3	2927.0	√	1.1%	-
	Worst	3038.8	2978.5	√	2.0%	-
	STD	23.8	25.6	x	-	7.1%
F10	Best	3098.5	3093.2	√	0.2%	-
	Mean	3116.2	3129.6	x	-	0.4%
	Worst	3203.9	3209.5	x	-	0.2%
	STD	23.1	36.4	x	-	36.5%

**Table 3:** TCSC device addition and their compensation levels for Case 1.

	Initial Case	ISAOA	SAOA	AEO	GWO	AQA	PSO
VG 1	1.0500	1.1	1.1	1.099351	1.099568	1.1	1.075
VG 2	1.0400	1.09755	1.1	1.094747	1.095818	1.1	1.07809
VG 5	1.0100	1.079716	1.1	1.074308	1.08001	1.09728	1.065148
VG 8	1.0100	1.08684	1.1	1.083873	1.085785	1.09288	1.063441
VG 11	1.0500	1.1	1.1	1.099959	1.078706	1.1	1.022355
VG 13	1.0500	1.1	1.1	1.099709	1.081997	1.1	1.038084
Ta 6-9	1.0780	1.067173	1.1	1.028284	1.025412	1.1	1.057744
Ta 6-10	1.0690	0.9	1.002677	0.925326	0.961107	0.910477	0.986684
Ta 4-12	1.0320	0.986297	1.010226	0.999935	1.008998	1.009181	1.002048
Ta 28-27	1.0680	0.973996	0.978184	0.98665	1.00225	1.034419	0.999681
Qr 10	0	5	5	4.152465	2.13309	5	2.240947
Qr 12	0	5	2.979705	4.930084	3.124115	3.962959	1.838463
Qr 15	0	4.999997	4.851496	4.952519	0.258411	5	0.558184
Qr 17	0	4.999982	4.624229	4.912524	3.793636	5	2.661792
Qr 20	0	4.081398	3.910989	1.71465	2.796705	5	3.442288
Qr 21	0	4.968112	5	4.899575	4.209032	5	3.753513
Qr 23	0	2.58453	2.684319	0.885251	3.763496	4.881004	3.735606
Qr 24	0	5	4.291999	3.534451	3.481095	5	3.049624
Qr 29	0	2.275642	2.726024	2.708482	2.864193	3.107001	1.325189
PG 1	99.2400	51.21077	51.5016	51.4936	62.3303	51.3952	54.40648
PG 2	80	80	80	79.78346	79.61742	80	80
PG 5	50	50	49.89148	49.86303	49.8189	50	48.99943
PG 8	20	35	35	34.99899	33.99505	35	34.5
PG 11	20	30	30	29.55887	29.7921	30	28.86584
PG 13	20	40	40	39.98309	37.77225	40	40
TCSC installed Lines	-	28-27	23-24	28-27	4-6	6-28	26
TCSC Compensation Percentage	-	−49.998%	+4.665%	−49.490%	−35.028%	−42.017%	−22.85%
Losses (MW)	5.832400	2.8217	3.061	2.844	3.035	2.990	3.37174

Positive and negative signs indicate an addition or subtraction in the transmission line reactance installed with a TCSC.

**Table 4:** TCSC device addition and their compensation levels for Case 2.

	Initial Case	ISAOA	SAOA	AEO	GWO	AQA	PSO
VG 1	1.0500	1.1	1.1	1.099352	1.095119	1.1	1.075
VG 2	1.0400	1.097584	1.1	1.096829	1.089415	1.1	1.075013
VG 5	1.0100	1.079817	1.1	1.078726	1.071614	1.1	1.042909
VG 8	1.0100	1.087006	1.1	1.086607	1.078618	1.094221	1.067203
VG 11	1.0500	1.1	1.1	1.099927	1.084584	1.1	1.075
VG 13	1.0500	1.1	1.047568	1.099558	1.074647	1.1	1.071508
Ta 6-9	1.0780	1.064807	1.042502	0.976591	1.049704	1.044803	1.02662
Ta 6-10	1.0690	0.900035	1.1	1.016372	1.032416	0.929538	0.959426
Ta 4-12	1.0320	0.980145	1.066829	1.00762	1.063337	1.011769	1.003132
Ta 28-27	1.0680	0.980535	1.066162	0.994596	1.002449	1.023534	1.010488
Qr 10	0	5	5	4.482774	3.497458	5	2.03322
Qr 12	0	5	5	3.665279	0.832066	5	3.70069
Qr 15	0	0	5	3.945993	4.166941	4.987603	1.505697
Qr 17	0	5	5	4.659533	3.173012	5	3.098415
Qr 20	0	5	5	4.934657	0.851722	4.879124	2.663595
Qr 21	0	4.999997	5	2.590238	3.394242	5	1.928459
Qr 23	0	4.274824	4.567337	2.648497	1.978594	5	3.428343
Qr 24	0	5	4.920762	4.935695	1.815333	5	4.035329
Qr 29	0	2.352175	3.409656	2.42653	0.977867	5	1.656887
PG 1	99.2400	51.18843	51.50164	51.49365	62.3303	51.39525	63.56449
PG 2	80	80	80	79.80634	72.62864	80	75.34649
PG 5	50	49.99428	50	49.99955	49.966	50	50
PG 8	20	35	35	34.98885	32.48052	35	31.89516
PG 11	20	30	30	29.99324	29.75128	30	29
PG 13	20	40	40	39.98501	39.47006	40	40
First TCSC installed Lines	-	28.27	6-28	6-9	6-8	10-17	26
First TCSC Compensation	-	−50.00%	3.72%	16.10%	24.83%	−13.64%	−50%
Second TCSC installed Lines	-	6-28	23-24	4-12	16-17	6-28	15
Second TCSC Compensation	-	−50.00%	16.57%	49.90%	−2.74%	−44.06%	6.417%
Losses (MW)	5.832400	2.820	3.102	2.867	3.227	2.995	3.360665

Positive and negative signs indicate an addition or subtraction in the transmission line reactance installed with a TCSC.

**Table 5:** TCSC device addition and their compensation levels for Case 3.

	Initial Case	ISAOA	SAOA	AEO	GWO	AQA	PSO
VG 1	1.0500	1.1	1.093023	1.099981	1.086747	1.097922	1.075036
VG 2	1.0400	1.1	1.091724	1.095393	1.082197	1.097226	1.076742
VG 5	1.0100	1.082327	1.071226	1.076605	1.061782	1.082092	1.047007
VG 8	1.0100	1.08939	1.088734	1.083436	1.068615	1.091701	1.054737
VG 11	1.0500	1.1	1.059316	1.088431	1.081636	1.095909	1.082164
VG 13	1.0500	1.1	1.049747	1.099988	1.070184	1.088444	1.074414
Ta 6-9	1.0780	1.1	1.056802	0.996884	0.993708	1.01364	0.973067
Ta 6-10	1.0690	0.9	0.992325	0.949036	1.042592	1.045173	0.993484
Ta 4-12	1.0320	0.990567	1.066913	1.031631	1.018614	1.063428	1.058055
Ta 28-27	1.0680	0.989463	1.048915	0.976953	0.991104	1.020301	0.971079
Qr 10	0	5	5	4.374692	2.966956	5	2.932858
Qr 12	0	1.5 × 10^−6^	4.893785	4.366721	0.713832	3.430701	4.286213
Qr 15	0	5	4.830902	4.974559	1.657207	1.754133	1.763583
Qr 17	0	5	4.93972	0.865704	1.784874	4.858897	3.538395
Qr 20	0	4.40039	4.97807	2.802873	2.792935	5	2.196743
Qr 21	0	5	4.983362	4.07292	1.804884	5	0.760047
Qr 23	0	2.71585	5	1.849487	1.079345	5	1.500662
Qr 24	0	5	4.999963	4.716259	3.888447	5	2.947927
Qr 29	0	2.271475	4.99007	2.050629	2.454247	5	2.648181
PG 1	99.2400	51.18571	51.37345	51.43609	56.25298	51.36892	58.62718
PG 2	80	80	80	79.97287	78.58037	80	76
PG 5	50	50	50	49.99684	49.96991	50	50
PG 8	20	35	35	34.99919	33.73529	35	35
PG 11	20	30	30	29.97878	28.5719	30	30
PG 13	20	40	40	39.89602	39.47651	40	37.10127
First TCSC installed Lines	-	6-28	6-28	28-27	9-11	10-17	9-10
First TCSC Compensation	-	−36.96%	2.06%	−44.65%	−0.62%	−39.76%	−8.80%
Second TCSC installed Lines	-	10-20	-	6-7	12-13	6-28	12-14
Second TCSC Compensation	-	−50.00%	-	−5.97%	−7.28%	6.83%	5.01%
Third TCSC installed Lines	-	28-27	-	10-20	-	25-26	4-12
Third TCSC Compensation	-	−50.00%	-	−49.50%	-	−50.00%	−24.71%
Losses (MW)	5.832400	2.821	3.036	2.880	3.187	2.969	3.3284477

Positive and negative signs indicate an addition or subtraction in the transmission line reactance installed with a TCSC.

**Table 6:** TCSC device addition and their compensation levels based on ISAOA for the three cases studied.

Control Variables	Initial Case	Maximum Compensation of 50%			Maximum Compensation of 70%		
		Case 1	Case 2	Case 3	Case 1	Case 2	Case 3
VG 1	1.0500	1.1	1.1	1.1	1.1	1.1	1.099999
VG 2	1.0400	1.09755	1.097584	1.1	1.097319	1.097956	1.097605
VG 5	1.0100	1.079716	1.079817	1.082327	1.078955	1.080331	1.07971
VG 8	1.0100	1.08684	1.087006	1.08939	1.086296	1.087668	1.087057
VG 11	1.0500	1.1	1.1	1.1	1.1	1.098732	1.1
VG 13	1.0500	1.1	1.1	1.1	1.1	1.099938	1.1
Ta 6-9	1.0780	1.067173	1.064807	1.1	1.063184	1.070089	1.067166
Ta 6-10	1.0690	0.9	0.900035	0.9	0.90001	0.9	0.956462
Ta 4-12	1.0320	0.986297	0.980145	0.990567	0.990751	0.98751	0.986089
Ta 28-27	1.0680	0.973996	0.980535	0.989463	0.988098	0.984445	0.983499
Qr 10	0	5	5	5	4.274806	5	0.108196
Qr 12	0	5	5	1.5 × 10^−6^	4.99999	4.956941	5
Qr 15	0	4.999997	0	5	5	4.709263	5
Qr 17	0	4.999982	5	5	5	4.996445	4.999989
Qr 20	0	4.081398	5	4.40039	3.702088	4.802683	4.877439
Qr 21	0	4.968112	4.999997	5	5	5	5
Qr 23	0	2.58453	4.274824	2.71585	5	2.494547	0
Qr 24	0	5	5	5	5	5	4.994352
Qr 29	0	2.275642	2.352175	2.271475	5	2.309083	2.443029
PG 1	99.2400	51.21077	51.18843	51.18571	51.22007902	51.22321	51.17528
PG 2	80	80	80	80	80	79.98913	80
PG 5	50	50	49.99428	50	50	50	50
PG 8	20	35	35	35	34.99961	35	35
PG 11	20	30	30	30	30	29.99852	30
PG 13	20	40	40	40	40	39.99559	39.99992
First TCSC installed Lines	-	28-27	28-27	6-28	28-27	6-28	28.27
First TCSC Compensation	-	−49.998%	−50.00%	−36.96%	−70.0%	−70.0%	−70.0%
Second TCSC installed Lines	-	-	6-28	10-20	-	28.27	8-28
Second TCSC Compensation	-	-	−50.00%	−50.00%	-	−57.6%	6.2%
Third TCSC installed Lines	-	-	-	28-27	-	-	6-10
Third TCSC Compensation	-	-	-	−50.00%	-	-	−70.0%
Losses (MW)	5.832400	2.8217	2.820	2.821	2.82731	2.80645	2.775199

Positive and negative signs indicate an addition or subtraction in the transmission line reactance installed with a TCSC.

## Data Availability

Not applicable.
